# Single-cell analysis reveals key roles for Bcl11a in regulating stem cell fate decisions

**DOI:** 10.1186/s13059-015-0778-y

**Published:** 2015-09-21

**Authors:** Ashley N Powers, Rahul Satija

**Affiliations:** New York Genome Center, New York, NY 10012 USA; New York University, Center for Genomics and Systems Biology, New York, NY 10012 USA

## Abstract

Cell-cycle fluctuations drive significant transcriptomic heterogeneity in murine hematopoietic stem cells. Additionally, deletion of *Bcl11a* alters the regulation of hematopoietic stem cell quiescence, self-renewal, and fate choice.

Please see related Research article: http://www.genomebiology.com/2015/16/1/178

In this issue of *Genome Biology*, Tsang and colleagues utilize the Fluidigm C1 microfluidic system to profile a total of 180 hematopoietic stem cells (HSCs) from mice of two different genetic backgrounds using single-cell RNA-sequencing (scRNA-seq) [1]. They leverage these data to interrogate the primary sources of expression variability in HSCs, and to better understand how cellular heterogeneity is affected by the deletion of *Bcl11a*. Their findings highlight multiple roles for this important regulator, and point to the power of single-cell transcriptomics for disentangling heterogeneous and developing systems.

## Heterogeneity in HSCs

Mammalian HSCs are capable of replenishing all blood cell types. Multiple studies have used the expression of cell surface markers to further separate the HSC compartment into various cell types and lineage states, which can subsequently be tested for functional differences. While this marker-based division has uncovered many distinct cell types and intermediate progenitors defining the mammalian immune system, it requires a biased preselection of individual surface proteins, and fails to characterize additional heterogeneity within each marker subset. As a result, the molecular definition of lineage-primed HSC subsets remains an active and exciting area of research for multiple laboratories. For example, multiple recent studies have identified subpopulations of HSCs whose offspring exhibit a strong bias towards myeloid differentiation [2, 3], but without a consensus on the optimal surface markers to enrich for these cells.

Single-cell transcriptomics offers a powerful alternative to characterizing cellular heterogeneity. By measuring the expression of thousands of genes inside an individual cell, scRNA-seq enables the computational inference of cell types, states, and regulatory programs, without prior knowledge [4]. Thus, single-cell technologies represent a promising tool for the evaluation of heterogeneous progenitor populations in hematopoiesis. Indeed, while previous studies have identified key regulators for myeloid and lymphoid development, such as the C2H2 zinc finger transcription factor B-cell CLL/lymphoma 11A (Bcl11a) [5], an unbiased characterization using scRNA-seq in HSCs could reveal a large set of regulators involved in early lineage specification. scRNA-seq studies have already resulted in significant new insights into similar developing systems, ranging from the blastocyst [6] to the distal lung [7].

## Cell-cycle progression drives HSC heterogeneity

Tsang and colleagues combined a series of targeted and unbiased analyses to uncover the drivers of transcriptomic heterogeneity in murine HSCs. Multiple analyses, including the identification of variable genes, hierarchical clustering, and principal component analysis, all pointed to fluctuations in cell-cycle state as the predominant source of cellular heterogeneity. By comparing their results with a database of stage-specific cell-cycle genes, the authors showed that they could assign individual cells to each of the four cell-cycle stages. Similar results have been reported across a variety of single-cell studies [8], and this finding enabled the authors to calculate the fraction of cells at each stage, a measurement reflecting the kinetics of cell-cycle progression. For example, with high reproducibility across replicate experiments, they identified that 28 % of *Bcl11a*^+/+^ cells were assigned to a G_0_/early G_1_ phase, potentially representing a quiescent or dormant subpopulation of HSCs.

When analyzing *Bcl11a*-deficient cells, the authors similarly found significant heterogeneity in cell-cycle progression. Intriguingly, they observed that these cells tended to be assigned (61 %) to the S and G_2_/M cell-cycle phases, a more than twofold increase compared with the wild-type controls. Consistent with this finding, knockout cells exhibited reduced levels of known HSC quiescence regulators, and signature genes associated with HSC self-renewal. The authors thus proposed that Bcl11a acts as a key regulator of HSC proliferation and maintenance, and validated this finding using staining for proliferation markers, as well as donor transplantation experiments. The collection of these analyses demonstrates an important lesson that is likely to hold true in many single-cell studies: although cell-cycle variation may represent an unsurprising source of cellular heterogeneity, changes in cycling kinetics may also reflect important differences in cellular activation state and fate potential.

## *Bcl11a* deletion reorganizes HSC identity and fate

After controlling for cell cycle effects, the authors discovered *Bcl11a*-deficient HSCs split into two distinct subsets, each of which was largely distinct from *Bcl11a*^*+/+*^ cells. Consistent with the authors’ previous findings that *Bcl11a*-deficient cells fail to undergo proper lymphoid development [5], both populations exhibited a strong depletion for the expression of genes associated with common lymphoid progenitors. Interestingly, these additional clusters exhibited expression patterns that were largely consistent with distinct myeloid lineages, and further analysis suggested that these groups may resemble megakaryocytic-erythrocyte progenitors (MEPs) and granulocyte-macrophage progenitors. Thus, the loss of *Bcl11a* is associated with the loss of lymphoid competence, and precocious myeloerythroid priming within HSCs.

The authors combine these findings with their previous analyses on proliferation kinetics in HSCs in order to propose an intriguing dual role for Bcl11a. Linking the proliferative nature of *Bcl11a*-deficient cells with observed changes in gene expression, they suggest that the deletion of *Bcl11a* leads to both the loss of lymphoid competence among HSCs, as well as an activated increase in proliferation resulting in myeloid priming, fate restriction, and the loss of self-renewal. Notably, the authors identify a subset of *Bcl11a*-competent cells that partly overlap with the MEP-biased subpopulation. These cells expressed the highest observed levels of CD41, a marker previously associated with myeloid-biased HSCs [2], thus hinting that the authors may have identified a similar primed subpopulation in this study. Further investigation, potentially with larger numbers of single cells, will help to tease apart the molecular basis of lineage priming in hematopoietic progenitors.

## Concluding remarks

By complementing existing studies that have uncovered extensive functional heterogeneity in HSCs, single-cell transcriptomics allows unbiased interrogation of molecular heterogeneity within and across progenitor populations. Tsang and colleagues find that expression variability in HSCs is largely driven by fluctuations in cell-cycle state, which may enable the identification of quiescent and activated progenitors. In addition, they discover extensive changes in population structure upon deletion of *Bcl11a*, suggesting new roles for this proto-oncogene in maintaining the self-renewal of lymphoid-competent HSCs. As the scale and throughput of single-cell technologies continues to grow, aided by exciting advances in droplet microfluidics [9, 10], among others, future studies will extend these findings to reveal the compendium of intermediate progenitor states, along with the key transcriptional networks, that govern mammalian hematopoiesis.

## Abbreviations

HSC: Hematopoietic stem cell
MEP: Megakaryocytic-erythrocyte progenitor
scRNA-seq: single-cell RNA-sequencing
